# Reach and predictors of effects during nation-wide dissemination of the universal parenting program All Children in Focus

**DOI:** 10.1186/s12889-023-16823-0

**Published:** 2023-10-18

**Authors:** Livia van Leuven, Johanna Engelbrektsson, Martin Forster

**Affiliations:** Department of Clinical Neuroscience, Division of Psychology, Karolinska Institutet, Nobels Väg 9, 171 65 Solna, Sweden

**Keywords:** Dissemination, Parent-training, Parenting, Predictors, Reach, Universal prevention

## Abstract

**Background:**

Parenting programs have the potential to improve population health, if widely disseminated. However, wide-scale dissemination is challenging. Also, more knowledge is needed of whether parenting programs are effective for the variability of families in the general population.

**Methods:**

This study aimed to investigate who the universal parenting program All Children in Focus (ABC) reaches when offered in routine care in Sweden. A second aim was to investigate if the outcomes were predicted by factors related to family background, group leader experience, and homework completion. Questionnaires were collected before and after ABC from 1420 parents. Hierarchical regression analyses were performed to examine predictors of disruptive child behavior, parenting practices, and satisfaction.

**Results:**

ABC was available in about 40% of Swedish municipalities and reached a fairly representative population sample, with the exception that fewer fathers than mothers participated. The examined predictors explained a small proportion of the variance in the outcomes (2.5, 3.5 and 14.7%, respectively). Still, the effect on disruptive child behavior was statistically significantly larger for parents born in Sweden, with higher education, and older children. The effect on parenting practices was also larger for parents born in Sweden, for mothers, and for those practicing homework more frequently. Most examined predictors showed no statistically significant association with child and parenting outcomes. Parents were generally satisfied with ABC and the significant predictors of satisfaction had little practical meaning.

**Conclusions:**

A fairly representative group of parents across Sweden were reached by ABC. Background variables, homework completion, and group leaders’ experience explained a small proportion of variance in the outcomes. Meanwhile, the slightly lower intervention effects found for preschool children and parents born abroad calls for further investigation, since even small differences in effects can have an impact at a population level. The study also points to the importance of stressing homework completion and to increase the reach of universal parenting interventions to some underrepresented groups.

## Background

Interventions focusing on parents are suggested as key in reducing the global burden of mental illness [1, 2]. Parents play a crucial role in supporting children’s socio-emotional development [3]. Parents’ behavior is one of the most important factors for children’s health both during childhood and for their future adult health, and thus important to target to prevent mental illness [4, 5]. Given the preventive potential of improving parenting at a societal level, two central issues to investigate are the reach of parenting programs, and how the effect of interventions differ according to participant or provider characteristics.

Parenting programs teach parents strategies in line with research of child health and well-being of how to handle challenging situations and strengthen parent–child relationships. Programs at different prevention levels have been developed. Targeted programs (i.e., selective or indicated prevention) aim to reduce symptoms of those experiencing clinical levels of problems or are at high-risk. By improving parenting practices, i.e., parents’ behaviors when interacting with their children, targeted parenting programs are for example effective in reducing disruptive child behavior [6], improving parental well-being [7] and reducing child maltreatment [8]. Universal programs instead aim to prevent *onset* of illness by reducing exposure to risk factors. For parenting programs, this is achieved through improving positive [9, 10] and reducing dysfunctional or abusive parenting practices [9, 11–14]. Universal programs are briefer and target ordinary parenting challenges and are thus aimed at all parents.

A major challenge is how to scale up interventions to reach parents with varying needs [1, 15, 16]. Making parenting programs available for *all* families is critical to attain several of the United Nations Sustainable Development Goals [1, 2]. Universal programs are suitable for the general population, of whom most do not experience clinical levels of problems. Also, universal programs can be less stigmatizing and reach those with negative attitudes towards treatment services [17]. Although policies and decades of research stress the benefits of parenting programs for public health, they have not been widely implemented in most countries [1].

Another challenge in large-scale dissemination is the generalizability of effects. RCTs often include samples not representative of the general population [18]. It is not certain that such effects will be generalized during wide-scale dissemination. In dissemination to the general population, a larger proportion of the population with more varying needs and characteristics are reached than when targeting certain groups. This gives the possibility to impact population health. Effect sizes which for high-need groups would be considered small can then have a substantial impact which justifies use of the program, requiring large sample sizes to detect [19]. Also, therapists in RCTs often have a higher degree of expertise in the interventions and systems to monitor adherence than providers in routine settings. A central question when a program is disseminated is thus whether it is effective across a variability of parents and providers. Given that smaller effects are meaningful for universal interventions, smaller differences in effects for e.g., different societal groups or different therapists are expectedly more meaningful as well.

Identifying whether the effect of interventions differ according to participant or provider characteristics can aid the understanding of whom an intervention works for, i.e., moderators or predictors of effects. This can be particularly relevant when an intervention is disseminated to the general population. Understanding variability of effects for families with different characteristics (e.g., socioeconomic differences) can guide families to the right support. Adjustments or extra support may be needed for those benefiting less. Studies have examined the predictive and moderating effect of a range of factors on the outcomes of parenting programs. The results remain mixed, but some predicting variables are more commonly established.

There have been inconsistent findings of whether family strain (e.g., low socioeconomic status (SES), poorer parental mental health) affects the outcomes of parental support. Earlier, well-cited meta-analyses have shown that SES-related disadvantages (e.g., low family income, education, occupation) reduce benefits [20–22]. Other aspects of strained or disadvantaged family situations have been associated with reduced benefits, such as mothers’ mental illness, single parenthood, larger family size [21, 22]. Meanwhile, recent meta-analyses have found the effects of parent training to be robust across varying levels of social disadvantage, including SES, education, and ethnic minority [23, 24].

Engagement can also impact outcomes. Studies have shown an association between higher engagement (e.g., sessions attended, completion of homework assignments, engagement in discussions) and benefits [25–27].

The influence of child characteristics is somewhat unclear. Some studies have found that parent training is more effective for younger than older children [28], while others have not [21, 29]. In most studies, the effect of parent training does not depend on child gender [29].

As for therapist background and experience, we only know of one meta-analysis that has included this dimension in the analysis. The study showed no moderating effect of therapists’ profession for programs in routine clinical settings [28].

A limitation with the published studies on predictors is that the majority have concerned targeted parenting programs, i.e., directed at sub-groups with higher needs; it is not certain that those findings can be generalized to universal programs. One of the few studies we know of predictors or moderators of universal parent training is an evaluation of the program in focus in this study: the Swedish *All Children in Focus* (‘ABC’, from the Swedish ‘Alla Barn i Centrum’). ABC is a universal four-session parenting program which has proven effective on parental self-efficacy and children’s health and development [30], which also has been shown to be possible to deliver both on-site and remotely [31]. Low parental mental health, higher education, and larger family size was associated with greater gains in parental self-efficacy in the intervention group compared to waitlist control. The other variables studied did not moderate parental self-efficacy (child age and gender, parental birth country). Regarding child health and development, low parental mental health and older children were associated with larger improvements. Neither education level, number of children, birth country, nor gender of child moderated child health and development [30].

### The present study

A purpose of universal parenting programs is prevention through dissemination at population level. The aim of the present study was to investigate who the ABC program reaches and if outcomes are predicted by factors relating to family background, group leader experience, and homework completion. While the RCT [30] investigated effectiveness under more controlled circumstances, this study explores factors that can influence outcomes when municipalities independently implement the program. This study was a part of an ongoing project evaluating the nation-wide dissemination of ABC through collecting routine assessments from parents taking part in the program over the country. The data included in the present paper was a sub-sample of the project’s larger data collection. Given that ABC is a universal prevention program aiming to reach all groups of parents, we used a threefold operationalization of reach: (1) the extent to which the parents who participated in ABC-groups were representative of the general population in Sweden, (2) the proportion of Swedish municipalities which offered ABC to parents, and (3) the diversity in background of the group leaders of ABC. The research questions were: (1) Which parents and group leaders does ABC reach within Sweden? (2) Do family characteristics, group leader experience, and/or parental homework completion predict program effects (disruptive child behavior and parenting practices) and satisfaction with ABC?

## Methods

### Procedure

ABC was disseminated over Sweden by a Train-the-Trainer approach. We trained trainers (henceforth ‘instructors’) who educated group leaders in municipalities across Sweden. Training of instructors took place once per year from 2017 to 2021. Each municipality was responsible for local implementation and maintaining program quality. They were encouraged to engage in quality assurance procedures and using guidelines and support material. Presentations, brochures and other material to market ABC were provided. To support the sustainability and quality of the implementation, we also provided yearly conferences for instructors, a support function through the project’s website, emails with updates, and statistics with preliminary data from the project. A future article will present more details on the Train-the-Trainer dissemination.

Data was collected from parents and group leaders all over Sweden. Almost all municipalities in Sweden offering ABC were invited to take part in the project. All ABC group leaders in Sweden were thus asked to invite parents in their groups to participate in the research project. Parents who gave consent to participate completed questionnaires online at the start (T1) and end of ABC (T2). Before administering the T1-questionnaire, group leaders gave verbal and written information to parents about the project. A minority of the parents responded on paper instead of online questionnaires at T1 and T2. In those cases, their group leaders entered their answers in the online system at a later time point. Unfortunately, the online system only registered the date of entry, and not the actual date that parents responded on paper. Thus, the range of days between T1 and T2 became possibly unreliable and sometimes obviously so (e.g., zero days). It was therefore not possible to reliably calculate the mean, standard deviation, and range of the number of days between T1 and T2. Instead, the median (42 days) and range between the 10^th^ and 90^th^ percentile (21–98 days) were calculated, excluding 51 obvious outliers. Parents declining to participate in the research took part of ABC in the same way as research participating families with the exception of not answering questionnaires.

### Participants

In total, *n* = 3446 parents completed the T1-questionnaire between autumn 2018 and spring 2021. Among those, *n* = 1420 completed the T2-measurement. Common reasons not to complete T2 was absence from the last session or that questionnaires were not distributed. No inclusion- or exclusion criteria were applied. For demographics of the families, see Table 1. At least 426 group leaders led the groups that the parents took part in. The study was ethically approved by the Regional Ethical Review Board in Stockholm, Sweden (registration numbers 2018/1661–31/1, 2021–03346).

**Table 1:** Background variables and baseline outcome data of the sample *N (%)* or *M (SD)*

	**Total sample** ***n* = 3446**	**Completers** ***n* = 1420**	**Non-completers** ***n* = 2026**	**F / X**^**2**^	***p***
***Parent characteristics***					
Mothers	2457 (71.3)	1018 (71.7)	1439 (71.0)	0.0	0.99
Education^a^					
Elementary school	142 (4.1)	37 (2.6)	105 (5.2)	14.0	**0.00**
High school/vocational school	1336 (38.8)	549 (38.7)	787 (38.8)	0.0	0.91
University	1830 (53.1)	781 (55.0)	1049 (51.8)	3.5	0.06
Other	138 (4.0)	53 (3.7)	85 (4.2)	0.5	0.50
Born in Sweden^b^	2795 (81.1)	1223 (86.1)	1572 (77.6)	39.7	**0.00**
***Characteristics of focal child***^***c***^					
Age	5.8 (2.7)	5.6 (2.6)	5.9 (2.7)	8.1	**0.01**
Girls	1030 (41.9)	427 (41.9)	603 (41.9)	0.0	0.96
Siblings living with child^d^	1.2 (0.9)	1.2 (0.8)	1.2 (0.9)	1.0	0.32
***Parent ratings***					
PP	24.5 (4.5)	24.3 (4.6)	24.7 (4.3)	3.9	**0.049**
SIDB	2.5 (1.9)	2.5 (1.9)	2.5 (2.0)	0.1	0.82

Significant variables in bold; Total sample = all who completed T1; differences between completers (those who completed both T1 and T2) and non-completers (did not complete T2) were examined with One-Way ANOVA (numerical variables) and Chi2 (categorical variables)

^a^In Sweden's general population aged 25–54 in 2021, 10% had successfully finished elementary school, 41% had completed high school or vocational education, and 49% had attained some level of university education [32]

^b^In 2021, 72% of the Swedish population aged 25–54 was born in Sweden [32]

^c^Child characteristics are presented for mothers ratings (71% of respondents) since we could not identify if parents belonged to the same family for all non-completers

^d^In 2021, among the children 0–17 years old in Sweden, 18% lived with 0 siblings, 48% with 1 sibling, 23% with 2 siblings and 11% with 3 + siblings [33]

### Measurements

Parents responded to questions about demographic data (at T1), including where they participated in ABC (which municipality), parenting practices (at T1 and T2), disruptive child behavior (at T1 and T2), satisfaction with ABC (at T2), and homework completion (by the question ‘How often have you worked on the exercises at home that the group leaders suggested?’ rated from 1 – ‘Single occasions or not at all’ to 5 – ‘Several times a day’; at T2). The instruments to assess disruptive child behavior and parenting practices were developed with the aim of creating short and comprehensive measures, considering feedback in a feasibility assessment before the research project. The conclusion from the feasibility test was that questionnaires needed to be kept short and wording easy to read to facilitate data collection on a national level, to avoid selection bias towards more highly educated and motivated parents. In addition, the questions were developed to suit a universal target group. Many established instruments are developed for clinical groups. The instruments were constructed based on clinical experience and inspiration from established instruments assessing parent and child well-being and behavior. See [31] for a previous report employing the instruments. Parents were asked to base their responses on their child who currently posed the greatest challenge for them as parents (henceforth ‘focal child’).

#### The Parenting Practices (PP) scale

Parents answered six questions about parenting practices over the last two weeks. Items were rated on a seven point Likert scale from ‘Never’ (coded as 1) to ‘Many times a day’ (coded 7), resulting in a range of 6‒42 with higher scores indicating greater use of parenting skills. Four questions pertained to positive parenting practices – praising the child, playing or doing a nice activity with the child, preparing the child for an activity, or talking calmly with the child despite being upset (e.g., “How often have you prepared your child for something challenging in the last two weeks?”). These four questions were inspired by the instrument Parenting Young Children (PARYC) [34]. The remaining two items concerned using unfavorable behaviors (negative parenting practices) – to nag or yell at the child (e.g., “How often have you yelled at your child in the last two weeks?”). The questions about negative behaviors were inverted when summarizing the scale. Cronbach’s alpha (α) of PP was 0.53 at T1 and 0.50 at T2. A guideline for acceptable α in non-clinical samples is α > 0.70 [35] but this is less applicable for scales with few items given that α automatically increases when items are added. The inter-item correlation can then be more informative, i.e., the correlation between items. Average inter-item correlations of 0.15 – 0.50 is acceptable [36]. Inter item range for PP was 0.01 – 0.55 (*M* = 0.20) at T1 and 0.00 – 0.57 (*M* = 0.18) at T2.

#### The Scale for Impairment of Disruptive Behavior (SIDB)

A four-item instrument was developed to assess the degree of impairment caused by disruptive behavior in different areas of life over the last two weeks. To assess a child’s level of psychological and social functioning in everyday life (within the four areas ‘school or preschool’, ‘home’, ‘with friends’, and ‘leisure activities’) is an important part of clinical assessments of children seeking mental health services. Parents considered the following statement applied to each of the four areas: “Has the child been fighting or disturbing in the last two weeks so it’s been a problem in …”. They responded on a four-point Likert scale ranging from ‘Not at all’ (0) to ‘Very much’ (3), resulting in a scale range of 0–12. Higher scores indicated more problem behaviors. Inter-item correlations and α were *r* = 0.22 – 0.57 (*M* = 0.33) and α = 0.65 at T1, and *r* = 0.18 – 0.45 (*M* = 0.26), α = 0.56. at T2.

#### Psychometric properties of the SIDB

A separate psychometric evaluation of the scale was also conducted using data from an RCT of two parenting programs [37]. Data was collected from 211 parents with 161 children. At baseline, parents responded to the SIDB and the Eyberg Child Behavior Inventory (ECBI) [38]. Presence of Attention-deficit/hyperactivity disorder (ADHD)-diagnosis and clinical severity of the child’s problems (rated by clinicians) were also recorded. The SIDB was rated during seven consecutive occasions from baseline. The internal consistency of the SIDB was acceptable (α = 0.66–0.69), while the test–retest reliability was good (*r* = 0.63) across seven weekly ratings. The convergent validity of the measure was moderate (*r* = 0.30 with ECBI and *r* = 0.29 with Clinical severity ratings). As a test of criterion validity, scores on SIDB for children diagnosed with ADHD were compared to those without diagnosis, which resulted in a large difference (*d* = 0.97, *t*(42) = 5.0, *p* < 0.0001). The corresponding analysis for ECBI-scores showed a moderate difference between children with and without diagnosis (*d* = 0.50, *t*(55) = 2.8, *p* < 0.01). In sum, the psychometric evaluation of the SIDB indicates adequate psychometric properties.

#### Satisfaction scale

Parents’ satisfaction with ABC and their child’s improvement was assessed with six questions at T2. Five questions came from the Therapy Attitude Inventory (TAI) [39], a 10-item instrument assessing satisfaction with services and skill development after an intervention (e.g., ‘My general feeling about the program I participated in…’). A sixth item was also included (‘How well do you think the group leaders conducted the meetings?’). Parents responded on a five-point Likert scale coded 1 – 5 with response alternatives such as ‘Considerably worse’ to ‘Greatly improved’. The range of the scale is 6–30 with higher scores indicating greater satisfaction. Inter-item *r* = 0.21 – 0.63, (*M* = 0.38); α = 0.79.

#### Group leader background

Group leaders answered questions about education, professional experience, and when and in which municipality they were trained.

### Group leader training and the intervention

#### Training of instructors

The training included five days of lectures and exercises, training a cohort of group leaders, and 2–3 h of individual supervision based on videos when training group leaders. Group leaders were eligible for instructor training after leading at least two ABC groups. The role of the instructors will be more thoroughly covered in a future article regarding the Train-the-Trainer dissemination.

#### Training of group leaders

The group leader training consisted of four and a half days of lectures and practical exercises. Group leaders also arranged a parent group and completed a writing assignment.

#### ABC

ABC consists of four group meetings comprising psychoeducative lectures, discussions and exercises of how to employ different parent practices. Meetings are two and a half hours, held by 1–2 group leaders and take place biweekly in groups of 5–10 parents. Parents are given homework and are encouraged to practise with their child between sessions. Every session centers around one of the following themes: 1) Showing love, 2) Being there, 3) Showing the way, and 4) Picking your battles. The aim of the program is for parents to learn parenting techniques that can help parents handle challenging situations with their children and contribute positively to their children’s development. For more details on ABC, see [40].

### Statistical analyses

Analyses were carried out with Jamovi version 1.6.23.0 [41] and R statistical program version 2022.07.1 [42]. To assess the reach of ABC, demographic data on parent and group leaders was compiled descriptively and parent characteristics were compared to population statistics. Also, differences at T1 between ‘completers’ (those who completed measurements at both T1 and T2) and non-completers (did not complete T2) were examined with χ2-test and One-Way ANOVA. Inter-item correlations and Cronbach’s alpha were calculated to estimate internal consistency. Repeated measures ANOVA and Cohen’s *d* was used to estimate T1-T2 change for SIDB and PP.

Hierarchical regression analyses were performed to predict if factors related to the families (Block 1), group leaders (Block 2), and homework completion (Block 3) could predict the effect on SIDB, PP and Satisfaction ratings. Change scores were used, instead of predicting scores at T2 with scores at T1 as covariate, to avoid regression artifacts (i.e., spurious effects) [43]. Conceptually similar variables were entered in separate blocks. Block 1 contained characteristics of the parent and focal child: mother or father, higher education (university) or not, parent born in Sweden or not, sex and age of the child, and number of siblings. Block 2 contained group leader experience: years providing help and support to parents, and number of held ABC groups. We did not include ‘profession’ since we expect the professions of group leaders to have similar levels of training relevant to supporting children and parents. Block 3 contained the parents’ ratings of homework completion.

Continuous predictors (i.e., age of child, siblings, group leaders’ experience, and homework completion) and outcome variables were standardized for the regression analyses. Categorical predictors were dummy coded (1 or 0) and not standardized. The beta coefficient (β) for linear predictors indicates change in the outcome expressed in standard deviations for an increase of 1 in the predictor. The coefficients for categorical predictors, change in the outcome expressed in standard deviations when the predictor is coded 1 instead of 0. An implication of this is also that it is not possible to directly compare an estimate of a linear predictor with a categorical predictor. To illustrate the impact of predictors on SIDB and PP, we calculated Cohen’s *d* for different levels of variables that were significant in the regression analyses.

When two caregivers in the same family responded, we randomly included one of their answers for the regression analyses. Consequently, the sample size was reduced from 1420 to 1269 parents. With 1269 participants, alpha = 0.05 and 9 predictors, the test had a power of 96% to detect a small effect size according to the standard of Cohen [44]. Due to missing values on predictors (mostly variables related to group leaders’ background), the sample in the regression analyses was 1067. Rates of missing data in parent rated outcomes (*n* = 1269) was 0.6%.

## Results

### The reach of ABC within Sweden

Aggregating parents' and group leaders' responses showed that ABC had been offered in about 40% of Sweden’s 290 municipalities during the data collection. The ABC-groups were offered in both rural and non-rural municipalities geographically spread all across Sweden.

For demographic data for the study sample and general population, and differences between study completers and non-completers, see Table 1. Among those who at least started ABC (*n* = 3446), 71% were mothers, 53% had a university education (about 49% in the general population [32]), and 81% were born in Sweden (72% in the general population [32]). Their focal child was *M* = 5.81 (*SD* = 2.68) years old, 42% were girls, and children had on average one sibling who they lived with. Having one sibling is the most common number of siblings for Swedish children [33].

There were some differences at T1 between completers and non-completers. A slightly higher proportion of non-completers than completers reported that their highest educational level was elementary school (5.2% versus 2.6%; *p* < 0.001). A higher proportion of parents among completers were also born in Sweden than non-completers (86.1% versus 77.6%; *p* < 0.001). Regarding differences at T1 on parent-rated questionnaires, completers scored slightly lower (*M* = 24.3, *SD* = 4.6) on PP than non-completers (*M* = 24.7, *SD* = 4.3; *p* < 0.05).

Among the 426 group leaders who held the groups, 298 responded to questions about previous experience. Common educational/professional backgrounds were preschool teacher (*n* = 104), social worker (*n* = 93), and special education teachers (*n* = 30). The remaining had a variety of educations within health care (e.g., nurse), psychology/behavioral sciences/sociology, or training without university degree in child care related areas. Most common workplaces were Family center (*n* = 75), social services (*n* = 73), school (*n* = 57), and preschool (*n* = 59). Most of the remaining worked within other municipal or child-health services. They had worked *M* = 6.5 (*SD* = 6.3) years at their current workplace and had in total 0–45 years (*M* = 11.2, *SD* = 9.7) of professional experience in supporting parents. The group leaders had held *M* = 5.1 (*SD* = 5.5) groups in ABC or a similar program. Among the group leaders, 17% were trained in 2011–2016, before the training-of-trainers project started. The remaining 83% were trained through training-of-trainers (2017–2021). The group leaders trained before the training-of-trainers project started were trained in at least 20 municipalities. During the project time, group leaders were trained in about 50 municipalities.

### Predictors of program effects and satisfaction

Standardized estimates and significance levels of regression analyses are presented in Table 2. Among the 1067 parents included in regression analyses, parents’ T1 score on PP was *M* = 24.5 (*SD* = 4.8) and *M* = 27.5 (*SD* = 4.3) at T2 (*F* = 477, *p* < 0.001; *d* = 0.67). The corresponding values for SIDB were *M* = 2.4 (*SD* = 1.9) at T1 and 1.7 (1.3) at T2 (*F* = 241, *p* < 0.001; *d* = 0.48). The average satisfaction score was 26.2 (*SD* = 2.5). The predictors explained 1.6–14.7% of the variation in the outcomes (*R*^2^, see Table 2). There were small differences in *R*^2^ between model 1–3 for SIDB (3.2 to 3.5% variance explained) and PP (1.6 to 2.5%). For satisfaction, the difference between model 2 and 3 was greater; the explained variance increased from 5.7 to 14.7%. Below, we present estimates of final models (referred to as “model 3” for each outcome), where all variables were included. Differences between models are also presented. No issues with multicollinearity were detected (VIF of predictors = 1.01–1.27; tolerance = 0.79–0.99). An investigation of outliers was conducted on the outcome measures using the 1.5*IQR-rule, showing a total of 26 outliers across the three outcome measures (SIDB, PP and satisfaction). As a sensitivity check, all regression analyses were run again, excluding the 26 outliers. The results were essentially unchanged, apart from the result that “mother” shifted from being a significant predictor of PP (*p* < 0.05), to a trend (*p* < 0.10).

**Table 2:** Estimates of hierarchical regression

		**SIDB**^a^			**PP**			**SAT**	
	Model 1	Model 2	Model 3	Model 1	Model 2	Model 3	Model 1	Model 2	Model 3
Variable	β	β	β	β	β	β	β	β	β
**Block 1**									
Mother	-0.004	-0.00	0.002	0.181**	0.183**	0.161*	0.310***	0.308***	0.227***
University	-0.145*	-0.144*	-0.146*	0.068	0.066	0.084	-0.204***	-0.206***	-0.140*
Country	-0.214*	-0.207*	-0.211*	0.256**	0.257**	0.297***	-0.397***	-0.402***	-0.255**
Girl	-0.009	-0.006	-0.006	0.015	0.017	0.017	-0.023	-0.023	-0.024
Age	-0.153***	-0.152***	-0.154***	0.024	0.024	0.039	-0.110***	-0.110***	-0.056
Siblings	0.008	0.010	0.009	-0.019	-0.018	-0.015	0.042	0.042	0.053
**Block 2**									
Years		0.006	0.006		0.031	0.030		0.014	0.011
Groups		-0.060	-0.060		-0.045	-0.049		0.023	0.011
**Block 3**									
HC			-0.009			0.086**			0.313***
**Model test**									
*R*^2^	0.032***	0.035***	0.035***	0.016*	0.018*	0.024**	0.056***	0.057***	0.147***
*F*	5.83	4.84	4.31	2.83	2.36	2.92	10.38	7.92	20.20
**Comparison**									
ΔR2		0.003	0.000		0.002	0.007**		0.000	0.090***
*F*		1.82	0.09		0.94	7.30		0.54	111.85

In each block, variables that are added are presented; all variables from previous models are kept in later models; SIDB = The Scale for Impairment of Disruptive Behavior; PP = Parenting Practices; SAT = parents' scores on the satisfaction scale; Model test = *R*^2^, F and *p*-value of each model; Comparison = Statistics of model 2 and 3 compared to the previous model; Mother = Parent a mother (1) or father (0); University = Parent with (1) or without (0) university education; Country = Parent born in Sweden (1) or not (0); Girl = Focal child a girl (1) or boy (0); Age = Age of focal child; Siblings = Number of siblings living with focal child; Years = Number of years group leader had experience helping and supporting parents (an average if two held the group); Groups = Number of groups group leader had held (an average if two); HC = Homework completion, i.e., how frequently the parent practised between sessions

^*^ *p* < .05

^**^ *p* < .01

^***^ *p* < .001

^a^a negative coefficient (reduction in SIDB) presents a desirable outcome, vice versa for PP and SAT

University education, being born in Sweden, and child age were significant predictors in model 3 of SIDB, as well as in model 1 and 2. Parents who had a university education reported a greater change on SIDB than parents without a university education (β = -0.146, *p* < 0.05). Parents born in Sweden reported a greater change compared to parents not born in Sweden (β = -0.211,* p* < 0.05). The analyses also showed an effect of the age of the focal child; β = -0.154 for each standard deviation increase in child age (*p* < 0.001). No other variables were significant in any model. The *R*^2^ of each model was significant at the level of *p* < 0.001. The *R*^2^ did not increase significantly when adding Block 2 and 3, which indicates that the first model (background variables compared to intercept) accounted for the significant *R*^2^.

Being a mother, being born in Sweden, and more frequently completing homework were significant predictors in the final model of PP. Mothers attained a larger improvement at T2 than fathers (β = 0.161, *p* < 0.05). Parents born in Sweden also had a larger improvement compared to parents born outside of Sweden (β = 0.297, *p* < 0.001). These two predictors were also significant in model 1 and 2. Adding homework completion in the final model showed that parents who scored higher on homework completion (practised more frequently) attained a larger improvement (β = 0.086 per standard deviation increase in homework completion, *p* < 0.01). The *R*^2^ of model 1 and 2 was significant at the level of *p* < 0.05 and of model 3 at *p* < 0.01. The *R*^2^ increased significantly when comparing homework completion in the final model (*p* < 0.01).

Being a mother, born in Sweden, university educated, and completing homework were significant predictors in the final model of satisfaction. Mothers were more satisfied than fathers (β = 0.227, *p* < 0.001) and parents who completed homework more frequently were more satisfied than those practicing less (β = 0.313, *p* < 0.001). Contrarily, parents with university education (β = -0.140, *p* < 0.05) and Swedish-born parents (β = -0.255, *p* < 0.01) were less satisfied with ABC. Being a mother, born in Sweden, and having a university education were also significant in model 1 and 2. In model 1 and 2, parents of older children were less satisfied (β = -0.110, *p* < 0.001), but the effect disappeared when adding homework completion in model 3. The *R*^2^ of each model was significant at the level of *p* < 0.001. The *R*^2^ significantly increased when adding homework completion in the final model (*p* < 0.001).

**Fig. 1:**
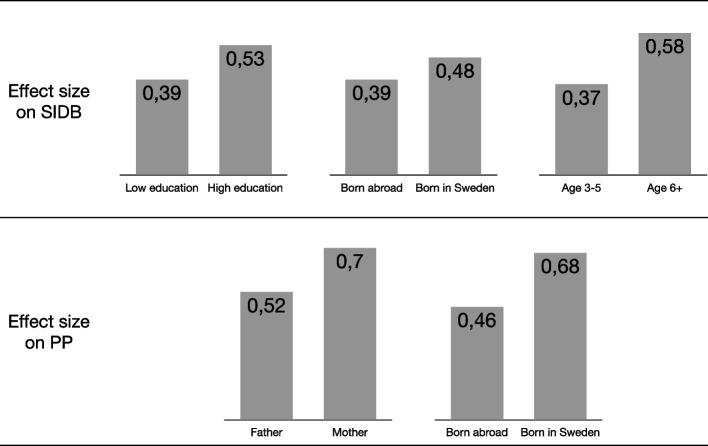
Effect sizes (Cohen’s *d*) for different levels of demographic variables that significantly predicted SIDB and PP T1-T2 effect sizes of significant predictor variables. SIDB = The Scale for Impairment of Disruptive Behavior; PP = Parenting Practices

**Fig. 2:**
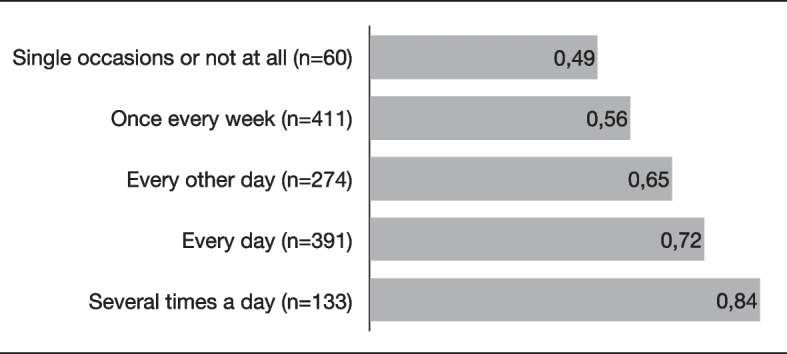
Effect sizes (Cohen’s *d*) on PP for different levels of homework completion PP = Parenting Practices

As illustrated in Fig. 1, effect size differences between levels of the significant demographic predictors (Block 1) ranged from *d* = 0.09 (effect on SIDB for parents born in Sweden vs. parents born abroad) to *d* = 0.22 (effect on PP for parents born in Sweden vs. parents born abroad). In Fig. 2, the effect size of PP is shown for each level of homework completion (Block 3). The difference in effect size between the two most extreme values (‘Single occasions or not at all’ vs. ‘Several times a day’) was *d* = 0.35.

Even if several demographic variables and homework completion were statistically significantly associated with satisfaction with ABC, the average satisfaction score (26.2) was close to the maximum score of 30 and the variation was low (*SD* = 2.5). Accounting for all significant demographic predictors, the least satisfied group of parents (fathers born in Sweden with a university education) had a mean satisfaction score of 25.2, while the most satisfied group (mothers born abroad without a university education) had a mean score of 27.6.

## Discussion

In this nation-wide dissemination of ABC, the program was offered in a large proportion of the municipalities in Sweden and reached parents that were fairly representative of the general population. Regression analyses showed that the examined predictors explained a small proportion of the total variance in the outcomes. This is expected for several reasons: First, the effect sizes of demographic predictors have in previous studies been in the small range [21, 22]. Second, in most other studies of predictors, the interventions have targeted risk groups, as opposed to the universal population included in this study. Since intervention effects generally are smaller in universal prevention [6], there is limited room to find strong effects of predictors. The intervention effect on SIDB was larger for parents who were born in Sweden, had higher education, and who had older children (school-age). The effect on PP was larger for parents born in Sweden and for mothers. The majority of the investigated variables were however not statistically significantly associated with outcomes. The level of homework completion was not significantly associated with SIDB, but showed a clear linear relationship with PP, with an incremental increase of effect for each level. Finally, parents were overall very satisfied with ABC and the statistically significant predictors of satisfaction had little practical meaning.

### The reach of ABC

A large number of group leaders were trained during the project, with the effect that ABC was offered in 40% of Swedish municipalities during the period of data collection. Municipalities over a large geographical area were represented. The sample of families was fairly representative of the general population regarding education level, birth country and family size. Group leaders from different educational backgrounds and workplaces were trained, which may have contributed to reaching families with different backgrounds. Many group leaders worked at preschools, which may be a particularly beneficial context to improve reach since over 90% of Swedish children attend preschools. The successful reach in geographical and demographical terms is promising given the benefits of large-scale dissemination of parent training [1, 2].

More mothers (71%) than fathers participated. This is quite typical for studies of parent training [45, 46]; reaching fathers is a well-known challenge, which our results point to. Meanwhile, increased father involvement have shown positive influence on outcomes [47].

It was more common to be a non-completer in the study for two groups of parents. Among parents born abroad, 70% were non-completers compared to 56% among parents born in Sweden (*p* < 0.0001). Among parents who only had completed elementary school, 74% were non-completers, while 58% were non-completers among parents with educational levels higher than elementary school (*p* < 0.0001). However, we do not know if these numbers reflect drop out from the parenting groups, or just drop out from measurements at T2. Parents who complete questionnaires in Swedish studies are more likely to be university educated and to be born in Sweden [48].

### Predictors of outcomes

The results indicate that parents who participate in ABC, regardless of background and despite some significant differences between groups, can expect around medium effect sizes on parenting practices and disruptive child behavior (Fig. 1). Although the predictors explained a small proportion of the total variance in the outcomes, especially for SIDB and PP, the significant predictors can still have practical implications. Having a university education and older children contributed to a larger decrease in SIDB. This result is similar to the RCT of ABC, where university-level education and child age moderated outcomes [30]. Reviews and meta-analyses of moderators/predictors have mainly studied treatments or selective/indicated prevention. Parents with higher education or SES have benefited more in some analyses [20–22] but not in others [23, 24]. One study found that higher SES was associated with greater gains for parents that experience less initial problems with child behaviors [20], which generally would be the case in universal prevention. The results of the present study support that children to parents with lower education may benefit less from parent training within the context of universal prevention.

In contrast to our study, the RCT of ABC [30] found a moderating effect for family size, but no effect for parent birth country. We have not found other studies of parent training which specifically examined country of birth as a predictor of effects. It could be important to further investigate if parents born abroad need extra support to retain in and benefit from interventions.

The effect on mothers’ ratings of their parent practices was larger compared to fathers’ ratings, although the difference between mothers and fathers no longer was significant when outliers were excluded. A meta-analysis of the Triple-P parenting program had similar findings [49]; the average effect on parenting for mothers was *d* = 0.77, compared to *d* = 0.51 for fathers. Interestingly, there was no difference between mothers’ versus fathers’ ratings of disruptive child behavior in the present study (*d* = 0.47 and *d* = 0.46, respectively), while mothers generally have been reporting much larger effects than fathers in studies of the universal version of the Triple-P program [11, 50, 51].

There were also small differences in effects on parent practices between adjacent levels of homework completion (Fig. 2). The lack of a significant association between homework completion and child behavior could possibly be explained by the fact that ABC is a universal parenting program and that the children in our sample showed low levels of problems to begin with (i.e., less room for variation). The studies we know of that have found engagement or homework completion to predict outcomes concerned targeted prevention or treatment [25–27]. Finally, group leader experience did not predict outcomes. This is in line with the one relevant study we have identified on the matter, which showed no effect of therapist background [28]. 

### Strengths and limitations

Strengths of this study relate to that a quantitative examination of a nation-wide dissemination was carried out in naturalistic settings with a large sample. The external validity is expected to be high. A large proportion of Swedish municipalities participated, data came from their routine work over several years, and no exclusion or inclusion criteria were applied. The amount of data enabled us to explore a range of predictors with high power. Achieving enough power is a major challenge when studying benefits of interventions for the population rather than clinical sub-groups [19].

Main limitations concern measurements and drop-out rates. We have no data on participation and completion of ABC (only of the study). The sample was still reasonably representative in relation to population statistics. Also, less than 50% completed T2, which can have implication for the validity of findings. Our prediction analyses primarily apply to study and program completers. We know little of why parents did not respond at T2. However, we evaluated the program in real-world conditions, which is also a strength; procedures that take a great deal of time and entail a high degree of control (e.g., persistently reminding parents to respond and attend sessions) could have limited the drop-out rate, but also resulted in a less naturalistic context.

Relating to lowering the burden on sites, we had limited data on outcomes and fidelity, the measures consisted of few items, and were not well-established. Also, families can attend universal programs for reasons other than disruptive child behavior; there could be program benefits not captured by the measurements. We had to keep data collection procedures to a minimum to ensure that not only the most motivated parents and group leaders completed surveys. Primarily, it was important to find a scale with few and relevant questions. The instruments were chosen since no established scales were considered suitable. Despite these efforts, there were large drop-out rates. Establishing effective data collection routines and assessing reasons to drop-outs could improve quality in future parts of the project.

### Implications for practice

Even if the differences in effect sizes across demographic groups were low in terms of conventional standards (Fig. 1), their practical meaning can be significant within the context of universal prevention and large-scale dissemination. Several authors have argued that Cohen’s benchmarks for small, medium and large effects cannot be universally applied [19, 52, 53]. Even Cohen [44] stated that conventions were illustrative and are dependent upon context and area of intervention. Universal interventions reach a greater proportion of the population and those exposed have more varying needs than targeted interventions. The effect is an average of high and low need individuals. Therefore, very small or small effects (according to Cohen’s standards) can be relevant to the public health [19]. A review of meta-analyses concluded that empirically based effect sizes for universal prevention programs on child externalizing behaviors would be *d* = 0.13 (small), *d* = 0.20 (medium), and *d* = 0.28 (large) [53]. An effect of *d* = 0.13 may thus impact public health during dissemination of universal programs. The foundation for that argument is that universal programs are less comprehensive and more easily disseminated than targeted programs, and therefore has the potential to reach a larger population at a reasonable cost.

Thus, even if differences in effects of ABC between demographic groups were small from an individual viewpoint (*d* = 0.09 to *d* = 0.22—see Fig. 1), the differences are important to consider from a public health perspective considering the ongoing effort to disseminate ABC across Sweden. Also, the relationship between homework completion and effects on parenting practices stresses the importance of implementing ABC with fidelity with special attention to homework assignments. Moreover, the lack of association between group leader experience and outcomes indicates that group leaders with varying experience can be recruited. Meanwhile, it must be considered that there was a great amount of unexplained variance in the outcomes. Other variables relevant to the variation in outcomes need to be explored.

That only 28% of the parents were fathers and that parents born abroad seem to drop-out to a greater extent have practical implications. Together with the results pertaining to the prediction of effects, this means that extra measures should be taken to attract, engage and adjust to the needs of the groups that seem to gain less. Especially, fathers, parents with lower education and with younger children, since those results have been replicated to a greater extent. The findings regarding parents born abroad are more uncertain, given the lack of studies on the topic.

To engage more parents, current efforts in Sweden to develop programs for immigrant parents are promising [54]. Important components for these parents can be to help parents enter new social networks, support language learning, and how to adjust to the new culture. Helping parents overcome practical barriers can be useful as well, e.g., increasing flexibility in how to participate. Providing ABC online could be a way to engage parents who have difficulties taking part in on-site meetings due to e.g., travel time, arranging a baby-sitter, or feeling uncomfortable in an on-site group [31].

### Future research

Since this is one of few studies that have investigated predictors of universal parent training, more research on this subject is needed. Since “born abroad” is a broad and heterogenous category of parents, further studies need to investigate the effects for subgroups of parents born abroad. That fathers attend parenting programs less is established in several studies, but more research is called for on causes and interventions to compensate for this inequality. To further explore fathers’ perceptions of parent training and reasons for non-participation or dissatisfaction could give valuable insights into how to increase engagement and attendance. It is also crucial to develop outcome measures with broader relevance for universal prevention (not just conduct problems). Also, with feasibility to prevent drop-out given the importance of large sample sizes to evaluate programs disseminated at scale [19]. More research including follow-up data of parent training in routine care is also needed.

## Conclusions

ABC reached a large proportion of Swedish municipalities. The participants were fairly representative for the general population, with the exception that fewer fathers than mothers were reached. The effects on parenting practices and disruptive child behavior were around medium in size, with small effect size differences due to demographic variables. Given the number of parents which may be reached by ABC, those differences could still be important. However, the participants in ABC reported meaningful positive change regardless of background (see Fig. 1), which supports the current strategy of universal dissemination. The results also support the dissemination of ABC through group leaders with varying levels of experience, since group leader experience had no significant effects on the outcomes. From a public health perspective, it is important to conduct more research on the differential effects and adjustments to the needs of parents who gain less. Moreover, the study points to the importance of stressing homework completion.

## Data Availability

The datasets supporting the conclusions of this article are not publicly available due to respect of participants’ confidentiality. De-identified data could however be available from the authors upon reasonable request. Please contact the corresponding author for such requests.
